# The Strength of the Medial Olivocochlear Reflex in Chinchillas Is Associated With Delayed Response Performance in a Visual Discrimination Task With Vocalizations as Distractors

**DOI:** 10.3389/fnins.2021.759219

**Published:** 2021-12-09

**Authors:** Sergio Vicencio-Jimenez, Giuliana Bucci-Mansilla, Macarena Bowen, Gonzalo Terreros, David Morales-Zepeda, Luis Robles, Paul H. Délano

**Affiliations:** ^1^Departamento de Otorrinolaringología, Hospital Clínico de la Universidad de Chile, Santiago, Chile; ^2^Department of Otolaryngology-Head and Neck Surgery, The Center for Hearing and Balance, Johns Hopkins University School of Medicine, Baltimore, MD, United States; ^3^Departamento de Neurociencia, Facultad de Medicina, Universidad de Chile, Santiago, Chile; ^4^Departamento de Fonoaudiología, Facultad de Medicina, Universidad de Chile, Santiago, Chile; ^5^Instituto de Ciencias de la Salud, Universidad de O’Higgins, Rancagua, Chile; ^6^Facultad de Medicina, Biomedical Neuroscience Institute, Universidad de Chile, Santiago, Chile; ^7^Centro Avanzado de Ingeniería Eléctrica y Electrónica, AC3E, Universidad Técnica Federico Santa María, Valparaíso, Chile

**Keywords:** delayed responses, working memory, otoacoustic emissions, chinchillas, olivocochlear, cognition

## Abstract

The ability to perceive the world is not merely a passive process but depends on sensorimotor loops and interactions that guide and actively bias our sensory systems. Understanding which and how cognitive processes participate in this active sensing is still an open question. In this context, the auditory system presents itself as an attractive model for this purpose as it features an efferent control network that projects from the cortex to subcortical nuclei and even to the sensory epithelium itself. This efferent system can regulate the cochlear amplifier sensitivity through medial olivocochlear (MOC) neurons located in the brainstem. The ability to suppress irrelevant sounds during selective attention to visual stimuli is one of the functions that have been attributed to this system. MOC neurons are also directly activated by sounds through a brainstem reflex circuit, a response linked to the ability to suppress auditory stimuli during visual attention. Human studies have suggested that MOC neurons are also recruited by other cognitive functions, such as working memory and predictability. The aim of this research was to explore whether cognitive processes related to delayed responses in a visual discrimination task were associated with MOC function. In this behavioral condition, chinchillas held their responses for more than 2.5 s after visual stimulus offset, with and without auditory distractors, and the accuracy of these responses was correlated with the magnitude of the MOC reflex. We found that the animals’ performance decreased in presence of auditory distractors and that the results observed in MOC reflex could predict this performance. The individual MOC strength correlated with behavioral performance during delayed responses with auditory distractors, but not without them. These results in chinchillas, suggest that MOC neurons are also recruited by other cognitive functions, such as working memory.

## Introduction

Sensory perception is not just a passive phenomenon but involves the active participation of organism (Yang et al., 2016). In fact, in natural or ecological situations, the changes in the sensory organs are highly influenced by the internal process of the nervous system. This ranges from changes in our spatial relation with the environment to shifts in our focus or sensory priority. In this sense, our actions, cognition, and perception are interrelated, coupled in a sensorimotor cycle (Buhrmann et al., 2013; Di Paolo et al., 2017). Therefore, to understand the phenomenon of perception, it is necessary to know how our internal states and cognitive processes are associated with our sensory pathways. This is how top-down control pathways present themselves as compelling research targets for developing a better understanding of the cognitive control of perception.

In the auditory system, the efferent pathways form a neural network including the auditory cortex and subcortical nuclei, such as the thalamus, inferior colliculus, superior olivary complex (SOC), and cochlear nucleus (Malmierca and Ryugo, 2011; Elgueda and Delano, 2020). Through these efferent pathways, signals from the cerebral cortex can reach the cochlea *via* the olivocochlear (OC) system, which originates in the SOC (Rasmussen, 1946). In this context, it has been proposed that in cognitive processes like selective attention, descending signals modulate sensory responses at different levels of the nervous system (Johnson and Zatorre, 2006; Fritz et al., 2007; Lauer et al., 2021). For example, models of visual selective attention in the presence of auditory distractors have demonstrated changes in neural activity at different levels of the auditory pathway, including cortical regions (Woldorff et al., 1993; Shomstein and Yantis, 2004), subcortical nuclei (Hernández-Peón et al., 1956), the auditory nerve and the cochlear receptor (Delano et al., 2007). These changes in the cochlear and auditory afferent system functions have been attributed to modulations by the auditory corticofugal pathways (Aedo et al., 2016; Terreros et al., 2016). For instance, it has been shown that KO mice which lack efferent activity perform poorly on selective visual attention tasks in the presence of auditory distractors (Terreros et al., 2016). Furthermore, estimates of auditory efferent function in chinchillas [assessed by measuring the medial olivocochlear (MOC) reflex strength] have shown to predict visual attention performance in the presence of auditory distractors (Bowen et al., 2020). This evidence is also supported by findings in humans, which have reported modulations of otoacoustic emissions, a measure of cochlear hair cells activity, during visual selective attention (Wittekindt et al., 2014; Dragicevic et al., 2019).

All this information strongly supports the idea that the auditory descending pathways suppress irrelevant auditory stimuli when the organism focuses its attention on another sensory modality (such as vision). However, given the relevance of the olivocochlear system in the regulation of auditory input signals, it is not difficult to imagine that its cognitive control is not exclusively limited to sensory selective attention. Then, it is plausible that the OC system is sensitive to a wide variety of cognitive phenomena. For example, in a recent study, Marcenaro et al. (2021) showed evidence that the MOC reflex strength is modulated during visual working memory in humans. In this context, we investigated whether the MOC strength was associated with the behavioral performance of delayed responses (more than 2.5 s after stimulus offset) during a visual selective attention task in chinchillas. We compared the MOC reflex with the performance in quiet conditions and in the presence of two different types of auditory distractors: broadband noise (BBN) and chinchilla distress vocalizations. These were chosen for their difference in ecological relevance, with vocalizations being an ecologically more significant signal. Thus, we expected to find a correlation between performance in the presence of distractors and the MOC reflex, with vocalizations also having greater effects than BBN.

## Materials and Methods

### Animals

We used a total of 19 adult male chinchillas (*Chinchilla laniger*, 4 ± 1 years of age) weighing between 500 and 700 g at the beginning of the behavioral training. Six animals were excluded from the analyses: three chinchillas dropped out of the training protocol due to health concerns, two others did not meet the behavioral criteria (see below), and in one it was not possible to perform adequate measurements of the MOC reflex. All chinchillas were housed in individual cages in a temperature and humidity-controlled room with a reverse light–dark cycle (lights on from 8 p.m. to 8 a.m.). In addition, they spent at least 3 h per week in an enrichment room, where they could exercise and bathe. They were given *ad libitum* access to water during the experimental period and were deprived of food, maintaining 85–90% of their previous *ad libitum* weight. All procedures were approved by the local Bioethics Committee (Animal Bioethics Committee, permit number 0844, Faculty of Medicine, University of Chile) and were performed according to the Care and Use of Laboratory Animals (National Research Council, 2011). These animals and raw data were used in our work published by Bowen et al. (2020). Here, we performed new analyses regarding late and the inter-trial time interval (ITI) responses to assess the cognitive processes associated with delayed behavioral responses.

### Medial Olivocochlear Reflex Measurement

The strength of the MOC reflex was assessed by comparing values of distortion product otoacoustic emissions (DPOAEs), measured at 2f1–f2, in the absence and presence of broadband-noise contralateral acoustic stimulation (CAS; Maison and Liberman, 2000). DPOAEs were recorded using an ER-10B+ microphone system (Etymotic Research) with 40 dB gain, amplified 10,000×, filtered between 0.1 and 10 kHz (Krohn-Hite, model 3323), digitized with a 40 kHz sampling rate, and stored for off-line analysis.

**FIGURE 1:**
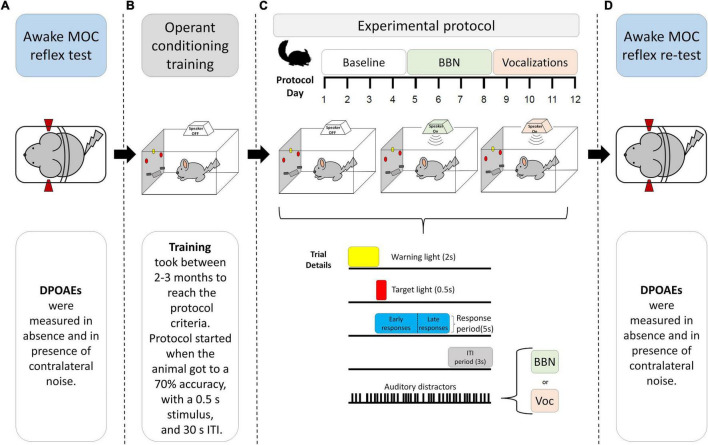
Experimental design diagram. The image schematizes the experimental tests to which the animals were subjected. First, the MOC reflex was evaluated in awake chinchillas with restricted mobility **(A)**. After the evaluation, training of the visual attention task was started in the operant conditioning apparatus **(B)**. After 2–3 months of training, the animals moved to the 12-day experimental protocol **(C)**, where they performed the task in silence and in the presence of auditory distractors (BBN and Chinchilla vocalizations). The time course of the visual discrimination task can be seen at the bottom of panel **(C)**. The task began with a central warning light (2 s), followed by the presentation of a side light (0.5 s) indicating which response lever was the target of that trial. The sidelight also initiated the response time (5 s) for the animal to press the lever and receive a reward. The last 3 s shown in the diagram corresponded to part of the ITI-time response period, where the animal did not receive a reward if it pressed the lever. Finally, at the end of the 12-day protocol, the MOC reflex of the Chinchillas was measured again **(D)**.

Tests to estimate the MOC reflex were performed on awake animals on two separate occasions (test and retest, Figures 1A,D). Awake chinchillas were carefully placed in a soft body and neck restraint, keeping the room temperature at 23–24°C and with the lights off. Before performing the DPOAEs measurements, the animals underwent at least three habituation sessions to the body restrictor, in which the time in the restrictor was gradually increased. On average, the chinchillas endured this restriction for about 30–40 min, and movements were monitored with a video camera inside the acoustic chamber. In cases of excessive movement and discomfort, the session was aborted. The test and retest were measured in two different weeks. These tests comprised 1440 trials divided into three blocks of 480 trials: before, during, and after CAS. All experiments were controlled with custom programs developed in C language (LabWindows).

The auditory stimuli with which DPOAEs were elicited consisted of seven ipsilateral primary tone frequencies, delivered to the right ear, where f2 was equal to = 1440, 2040, 2884, 4080, 5769, 6125, and 8160 Hz. On the other hand, the contralateral BBN used for CAS had an intensity of ∼60 dB SPL and was delivered to the left ear. Both stimuli were digitally generated using two synchronized PCI cards (6.071-E, National Instruments) at 100,000 samples/s, attenuated with PA-5 programmable attenuators (System 3, Tucker-Davis Technologies) and delivered through ER-2 transducers (Etymotic Research) sealed to both external auditory meatus and pinna. Primary tones were presented at a frequency of 4 Hz with a duration of 15 ms, a rise/fall time of 5 ms, a fixed ratio of f2/f1 = 1.25 and L1/L2 = 65/60 dB SPL, with a delay of 200 ms. Contralateral non-continuous BBN (0.2–10 kHz) was administered at a presentation frequency of 4 Hz with a duration of 170 ms. At the beginning of each experiment, the sound pressure level in both ears was calibrated with an Etymotic^®^ microphone.

### Behavioral Apparatus and Training Procedures

The behavioral task was performed in an operant conditioning apparatus identical to the one used in Delano et al. (2007), located inside a double-wall room that attenuated sound. The training procedures were performed by experimenters who were blind to the MOC reflex values of the chinchillas. The time that was required for training (from the start to the entry into the experimental protocol) was approximately 2–3 months (Figure 1B). First, the animals had 1–2 weeks of habituation to the chamber and then began their training sessions. The chinchillas trained one session per day, 5 days per week. The task consisted of a two-choice visual discrimination test, which we have previously used in rats (Hamame et al., 2006), chinchillas (Delano et al., 2007; Bowen et al., 2020), and mice (Terreros et al., 2016; Jorratt et al., 2017). The operant conditioning chamber was located inside a double-wall room that attenuated external sound. The front panel of the apparatus had a central light (warning signal) located above the food dispenser and two sidelights (targets), each located above one of the response levers (right and left). A trial began with the onset of the central light (warning period) that lasted for 2 s, followed by the random onset of one of the target lights for a period of 0.5 s. Chinchillas were trained to respond by pressing the corresponding lever under the lateral light during the 5 s response period from the onset of the target light (Figure 1C). The ITI period varied randomly between 27 and 33 s. Correct responses during the response period were rewarded with a 45 mg pellet (Noyes PJNI-0045 Chinchilla Food Pellet; Research Diets, New Brunswick, NJ, United States). Early responses (pressing during the central light period), incorrect responses (pressing the opposite lever during the response period), and ITI responses (pressing after the response period was over) were punished with a 40-s time-out period, during which all lights were turned off. Trials in which chinchillas did not respond were defined as omissions and did not receive a time-out punishment. The behavioral variables measured were accuracy [correct responses/(correct responses + incorrect responses)], number of correct and incorrect responses, the number of omitted trials, and the latency of the lever press (time between the onset of the target light and the lever press). During the training period, the number of trials per session, the duration of the target light, the ITI period, and the punishment time were progressively modified according to the performance of the animals. After the chinchillas achieved an accuracy of at least 70% during a session of 110 trials with protocol values of 0.5 s target light duration, ITI of 27–33 s, and punishment of 40 s, they were recruited for the first day of the experimental protocol.

### Experimental Protocol

The behavioral protocol consisted of 12 days of behavioral tests divided into three stages of 4 days with 110 trials each (Bowen et al., 2020; Figure 1C). On the first 4 days (baseline period), the chinchillas performed the two-choice discrimination task without auditory distractors, with the same parameters under which they finished the training stage. On days 5–8, the chinchillas performed the same visual discrimination task but in the presence of BBN as an auditory distractor (Figure 1C). Finally, on days 9–12, the chinchillas performed the visual discrimination task in the presence of an auditory distractor that consisted of alarm vocalizations from a male chinchilla (VOC) (Figure 1C).

### Auditory Distractors

As mentioned above, we used two different auditory distractors during the experimental protocol: (1) a BBN (0.02–20 kHz) as an ecologically irrelevant distractor and (2) male chinchilla vocalizations as an ecologically relevant distractor. All vocalizations were previously recorded in a distress context and published by Moreno-Gómez et al. (2015). We used four clean harmonic male vocalizations (one for each of the 4 days with VOC) with the fundamental frequency (F0) between 538 and 861 Hz and dominant frequency around 1200 Hz. BBN and VOC distractors were presented binaurally at ∼65 dB SPL through a speaker (Sony, frequency response 20–20,000 Hz) located 1 m above the operant apparatus in free field conditions. Auditory distractors were delivered at an irregular rate centered at 2.5 ± 1.0 Hz (1.5–3.5 Hz, pseudo-randomly distributed) to prevent or diminish habituation.

### Data Analysis

For the purposes of this study, we consider working memory as the transient representation of a signal during a period when the signal is no longer present and which serves to provide a subsequent response (Gazzaley and Nobre, 2012). Therefore, to study working memory, the response period was analyzed by looking at early and late time windows. The distinction between early and late time windows was determined by performing a frequency histogram for the latencies of the lever pressed. Using the temporal distribution of responses, the mean and standard deviation were calculated. With these parameters, we estimated the latency value that was more than two standard deviations away from the mean of the responses. That value was considered as the boundary between early and late responses. To estimate whether the average accuracy of animals during the basal period [the first stage of the behavioral protocol (Figure 1C)] was significantly greater than expected by chance, one-sample Student’s *t*-test was performed against an expected mean of 0.5. A mixed effect analysis considered potential changes in the accuracy of animal responses on different days and conditions (Baseline, BBN, and VOC) during the experimental protocol. *Post hoc* analysis was performed using a Dunnett multiple comparisons test. The association between MOC reflex strength (CAS-induced DPOAE changes) and behavioral performance for each stage of the behavioral protocol was separately assessed using generalized linear models. The data was fitted using binomial family with a logit link was used. These procedures were like the ones used by Bowen et al. (2020). Data processing and statistical analyses were performed with MATLAB and GraphPad prism. Within the figures, the error bars correspond to the standard error of the mean (SEM). Statistical significance was defined as: *p* > 0.05 not significant (n.s.) and *p* < 0.05 as significant.

## Results

**FIGURE 2:**
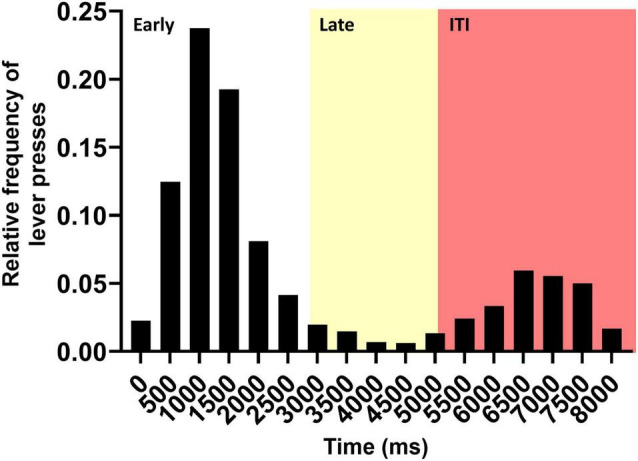
Histogram showing the frequency of the responses during the baseline period of the protocol (days 1–4). The image shows the relative frequency of lever presses as a function of the trial elapsed time. Each bin of the histogram corresponds to a 500-ms window. The zero represents the beginning of the animals’ response time (lateral light onset). The first 5 s represent the time in which the animal received a reward if it pressed the correct lever, which was separated into an early response window (white) and a late response window (yellow). The last 3 s correspond to the time in which the animal did not receive a reward for pressing the correct lever (red).

In this research, we evaluated the link between the MOC function and delayed responses in a visual discrimination task. For this, we observed the distribution of responses during the basal period of the protocol (Figure 2). We found that within the response period (0–5 s) the average lever press latency was 1.4 s with a standard deviation of 0.8 s. With this information, we defined the late responses as all those that were more than two standard deviations away from the mean latency. Thus, early responses corresponded to values between 0 and 3 s and late responses to values between 3 and 5 s from the lateral light onset (Figure 2, white and yellow, respectively). Late responses corresponded to approximately 5% of all lever presses. This time (at least 2.5 s post stimulus offset) is within the range of animal models of working memory (Wallace et al., 1980; Porritt and Poling, 2008; Lind et al., 2015). Along with this, when we looked at the totality of responses, we could see that ITI responses after the reward window were distributed between 5 and 8 s. No reward was received during ITI time, however, we found them interesting to analyze since they were made after receiving the lateral light stimulus, so the animal could potentially be making a late response to the signal. These responses could also involve working memory, considering that 4.5–7.5 s had elapsed after the stimulus offset. Thus, performance in these ITI responses (Figure 2, red) were also analyzed throughout the protocol.

**FIGURE 3:**
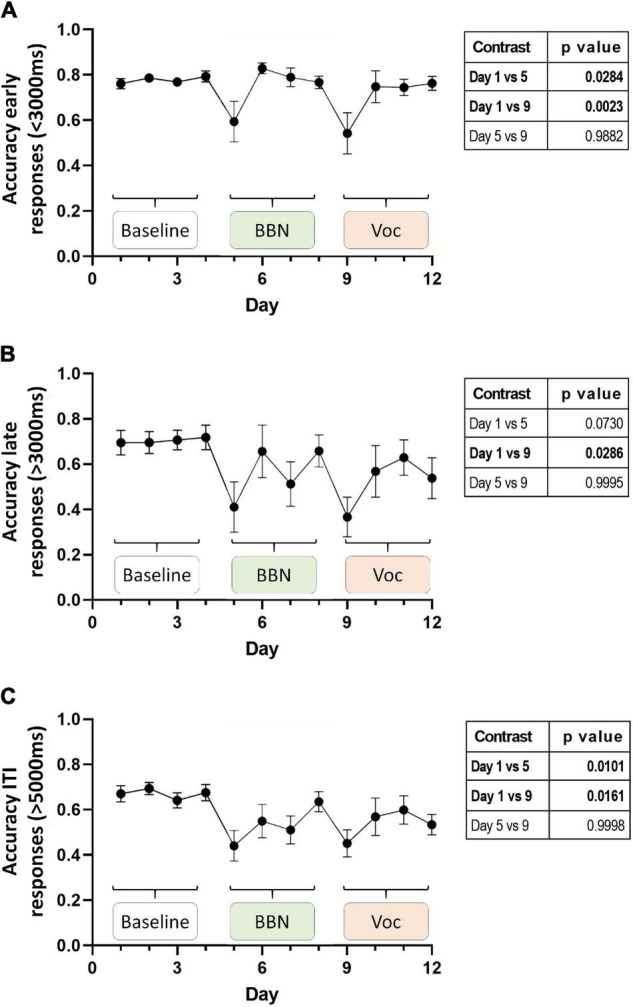
Average accuracy of behavioral responses during the 12-day experimental protocol. Panel **(A)** shows the average accuracy of the responses from the early time window (0–3 s). In panel **(B)** the average accuracy of the responses of late time window (between 3 and 5 s), while in panel **(C)** the average accuracy of the ITI responses (between 5 and 8 s) is shown. Data are displayed as mean ± SEM (*n* = 13 chinchillas). Using linear mixed effect models, we found significant effects for behavioral performance on days 5 and 9 (see section “Results” in main text). A table with *p*-values from the Dunnett multiple comparisons test is shown to the right of each figure.

Figure 3 shows the average performance of the animals during the 12-day protocol for each of these periods (Figures 3A–C, early, late, and ITI, respectively). As expected, during the basal period (days 1–4) of the early time window the chinchillas displayed high accuracy, averaging values close to 80% (0.78 ± 0.06). A mixed effect analysis found significant changes in accuracy throughout the experimental protocol [*F*(11,132) = 3.954, *p* < 0.0001], where a Dunnett’s *post hoc* test found significant decreases for days 5 and 9 (Figure 3A, table insert). The decreases for these days, which corresponded to the first days of auditory distractors (BBN and vocalizations, respectively) are in line with those reported previously by Bowen et al. (2020). Inspection of the performance in the late time window also showed high accuracy in the responses during the basal period. In these 4 days, the animals averaged an accuracy of 0.7 ± 0.17. This is consistent with the hypothesis that the animals are using working memory during this period. In this time window, a mixed effect analysis also found significant changes in accuracy during the experimental protocol [*F*(11,128) = 2.186, *p* = 0.0189], where Dunnett’s test found significant decreases only for day 9 of the protocol (Figure 3B, table insert). In addition to the above, we found that the results in the ITI-time window followed a similar pattern to those found in the response window period (Figure 3C). On average, 67% of the animals’ ITI responses during the basal period coincided with the lever signaled by the target light. In other words, if these responses had been within the reward window, the animals would have had 0.67 ± 0.1 accuracy. Moreover, these values differed significantly from the 0.5 accuracy expected only by chance [one sample *t*-test: *t* = 7.334, df = 12, *p* < 0.0001]. This evidence also implies that, even for these very late responses, chinchillas are using their memory to respond. Additionally, a mixed effect analysis found significant changes in accuracy during the experimental protocol [*F*(11,131) = 2.993, *p* = 0.0014], where Dunnett’s *post hoc* test identified a significant decrease in responses associated with the correct lever on days 5 and 9 of the protocol (Figure 3C, table insert).

**FIGURE 4:**
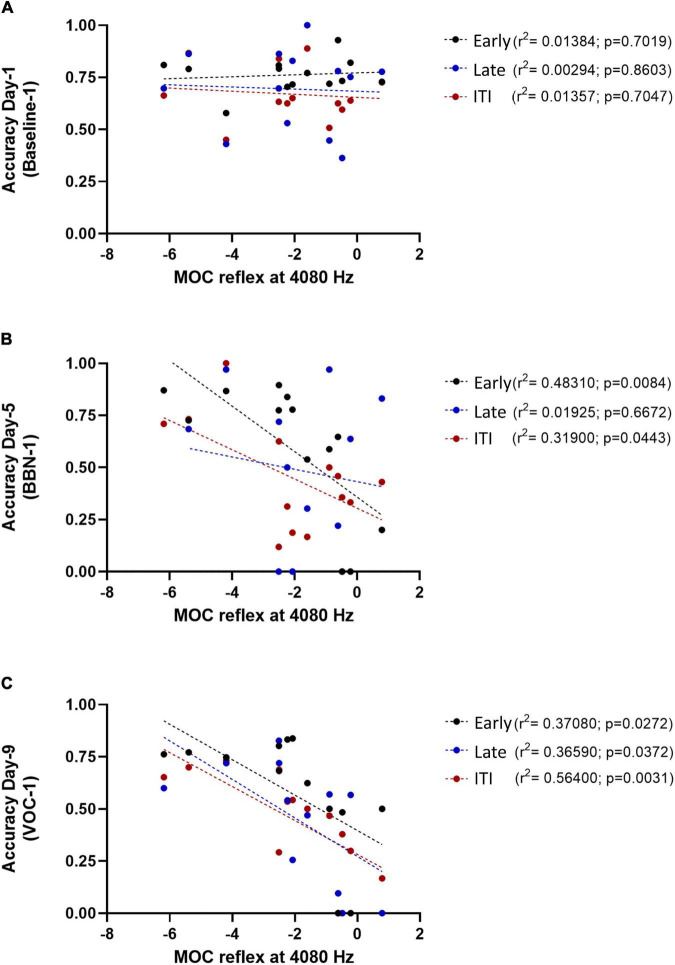
Association between the MOC reflex (at 4080 Hz) and individual behavioral performance on different days of the behavioral protocol. The panel **(A)** represents the accuracy values on day 1 of the protocol (Baseline-1) as a function of the MOC reflex at 4080 Hz. In panel **(B)** it is shown the accuracy values on day 5 of the protocol (BBN-1) as a function of the MOC reflex at 4080, while panel **(C)** shows the values for day 9 of the protocol (VOC-1). The circles represent the individual values for each Chinchilla (*n* = 13) and the dotted lines correspond to the fitting curve. Black circles correspond to early responses, blue ones to the late response and red circles to ITI responses.

With these results, we investigated the association between performance in the visual discrimination task with auditory distractors and a measure of the MOC reflex. In the same way as Bowen et al. (2020), we used MOC reflex strength values obtained from awake chinchillas and evaluated the correlation of these values with the animal’s performance at the three different periods during the behavioral protocol of 12 days. It is relevant to note that, in the absence of auditory distractors (during the baseline days), the MOC reflex was not a predictor of performance in any of the time windows (Tables 1–3 and Figure 4A).

**TABLE 1:** The generalized linear models evaluating the association between MOC reflex strength (DPOAE CAS-induced changes) and behavioral performance for early responses (<3 s).

Protocol day	Chi^2^-statistic vs. constant model	DPOAE Frequency (Hz)	Estimate	SE	*t*-Stat	*p*-Value
Day 1 (Baseline-1)	5.03, *p*-value = 0.284	2884	−0.006233	0.030897	−0.20172	0.84013
		4080	0.057822	0.039265	1.4726	0.14085
		5768	0.03734	0.044147	0.84582	0.39765
		6125	−0.02995	0.052086	−0.57501	0.56529
**Day 5 (BBN-1)**	**32.8, *p*-value = 1.15e−05**	2884	0.01303	0.053587	0.24316	0.80788
		**4080**	**0.23955**	**0.048723**	**4.9165**	**8.812e−07**
		5768	−0.061187	0.059469	−1.0289	0.30353
		6125	−0.040424	0.067286	−0.60078	0.54799
**Day 9 (VOC-1)**	**18.4, *p*-value = 0.0101**	2884	0.053524	0.032191	1.6627	0.096375
		**4080**	**0.15633**	**0.045017**	**3.4728**	**0.0005151**
		5768	0.0068814	0.050159	0.13719	0.89088
		**6125**	**−0.1753**	**0.058562**	**−2.9934**	**0.0027593**

*Significant results (p < 0.05 are bolded).*

**TABLE 2:** The generalized linear models evaluated the association between MOC reflex strength (DPOAE CAS-induced changes) and behavioral performance for late responses (3–5 s).

Protocol day	Chi^2^-statistic vs. constant model	DPOAE Frequency (Hz)	Estimate	SE	*t*-Stat	*p*-Value
Day 1 (Baseline-1)	4.63, *p*-value = 0.327	2884	0.045465	0.131	0.34705	0.72855
		4080	−0.20551	0.15893	−1.293	0.196
		5768	−0.05293	0.20972	−0.25239	0.80074
		6125	0.30701	0.24406	1.258	0.20841
Day 5 (BBN-1)	5.04, *p*-value = 0.655	2884	0.24536	0.16866	1.4548	0.14574
		4080	−0.24856	0.2133	−1.1653	0.24389
		5768	−0.21825	0.30756	−0.70959	0.47796
		6125	0.48615	0.40453	1.2018	0.22945
Day 9 (VOC-1)	4.68, *p*-value = 0.699	2884	−0.00335	0.10564	−0.03175	0.97467
		4080	0.2029	0.09824	2.0653	0.038895
		5768	0.074641	0.13236	0.56393	0.5728
		6125	−0.18391	0.19457	−0.94521	0.34455

**TABLE 3:** The generalized linear models evaluating the association between MOC reflex strength (DPOAE CAS-induced changes) and behavioral performance for ITI-time responses (>5 s).

Protocol day	Chi^2^-statistic vs. constant model	DPOAE Frequency (Hz)	Estimate	SE	*t*-Stat	*p*-Value
Day 1 (Baseline-1)	3.64, *p*-value = 0.456	2884	−0.04567	0.048928	−0.93344	0.35059
		4080	0.017122	0.057903	0.2957	0.76746
		5768	0.055745	0.076765	0.72618	0.46773
		6125	−0.07975	0.09046	−0.88169	0.37795
**Day 5 (BBN-1)**	**18, *p*-value = 0.00124**	2884	−0.01456	0.062792	−0.23194	0.81658
		**4080**	**0.20648**	**0.080313**	**2.571**	**0.010142**
		5768	−0.13803	0.093076	−1.4829	0.13809
		6125	−0.12727	0.10404	−1.2233	0.2212
**Day 9 (VOC-1)**	**93, *p*-value = 3e−19**	**2884**	**0.74137**	**0.12628**	**5.8709**	**4.333e−09**
		**4080**	**0.5084**	**0.15067**	**3.3742**	**0.0007402**
		5768	0.14837	0.16142	0.91913	0.35803
		**6125**	**−1.0817**	**0.2384**	**−4.5373**	**5.699e−06**

*Significant results (p < 0.05 are bolded).*

In the case of early responses (0–3 s) we found values very similar to those we previously reported (Bowen et al., 2020), identifying an association between the strength of the MOC reflex and the accuracy of the animals in the first days of the auditory distractor presentation (Table 1). For day 5 (BBN-1) the DPOAE amplitude at a frequency of 4080 Hz significantly correlated with task accuracy, while for day 9 (VOC-1) we found significant values for frequencies of 4080 and 6125 Hz (Table 1). On the other hand, analysis with generalized linear models for the late responses (3–5 s) found no significant link between the MOC reflex strength at different frequencies and the performance of the animals in the behavioral task (Table 2). In contrast, in the case of the ITI-time window (>5 s) we did find significant correlations between the strength of the MOC reflex and the accuracy of the animals on the first days of auditory distractor presentation (BBN-1 and VOC-1) (Table 3). We found that on day 5 (BBN-1) the DPOAE amplitude at a frequency of 4080 Hz correlated significantly with task accuracy, while for day 9 (VOC-1) the DPOAE at frequencies of 2884, 4080, and 6125 Hz were significantly associated with task accuracy (Table 3).

Figure 4 shows an example of the above mentioned correlations. It shows the individual values of accuracy in the three temporal windows as a function of the MOC reflex strength at 4080 Hz, for day 1 (Baseline-1, Figure 4A), day 5 (BBN-1, Figure 4B), and day 9 (VOC-1, Figure 4C). We chose the MOC reflex at this frequency because it was the one that presented the most significant associations with the behavioral results.

## Discussion

In the present work, we show that the functioning of MOC reflex, measured by the magnitude of DPOAEs suppression produced by contralateral noise in awake chinchillas, is associated with behavioral performance in a visual discrimination task with auditory distractors during periods in which working memory is relevant to accomplish the task. Specifically, we found that individual variability in the MOC reflex strength correlates with the accuracy of delayed responses (late and ITI responses, executed at more than 2.5 s from the target stimulus offset) in a visual discrimination task that was performed with chinchilla distress vocalizations as auditory distractors.

Our data were part of the same set used in a previous publication (Bowen et al., 2020), where we showed that the MOC reflex was a predictor of selective visual attention performance in the presence of auditory distractors. Here, we performed new analyses, including different periods in the behavioral task, which allowed us to study delayed responses. We divided correct responses into two periods: an early period (less than 2.5 s post target offset), and a late period (2.5–4.5 s post target offset). We also analyzed a third period of ITI responses, occurring 4.5–7.5 s after target offset, but with no reward and time-out punishment. The purpose of this separation was to have correct responses that were more related to the visual selective attention processes (early period), and responses that are probably related to visual and/or executive working memory (late correct and ITI). It is important to highlight, that in the cases of delayed responses (late correct and ITI), for having a good accuracy of discrimination, the visual target stimulus needed to be held for a few seconds in the memory buffer of the behaving chinchillas, indicating the use of visual, executive, or other type of working memory resource. As expected, the results in the early window were equivalent to those found by Bowen et al. (2020). Auditory distractors significantly decreased performance (Figure 3A) and MOC reflex values were good predictors for individual chinchilla performance (Table 1 and Figure 4).

On the other hand, it is relevant to note that the results obtained in the late and ITI time window were like those we observed in the early window and, therefore, to those reported by Bowen et al. (2020). We observed that, in the basal response period of the experimental protocol, both in the late and ITI time window, the animals’ correct responses were significantly greater than those expected by chance. Importantly, these results suggest that memory mechanisms are operating for late and ITI responses, allowing the animal to correctly press the lever associated with the brief target stimulus (0.5 s) that had disappeared more than 2.5 sec earlier. Furthermore, in these time windows we also found significant effects of auditory distractors on the animals’ performance. For the late window, we only found a significant decrease for the first day of VOC distractor (day 9, Figure 3B). Potentially this is because, unlike BBNs, vocalizations are ecologically relevant signals, so they are expected to have a greater distracting effect. However, given the trend that can be observed in the data and how close it was to statistical significance, we believe that the failure to find significant changes on day 5 of the late window was probably due to the low number of trials we had within this window. For the case of the ITI-time window, we found significant decreases for both the first day of BBN (day 5) and the first VOC day (day 9, Figure 3C).

With these findings, we looked at whether the strength of the MOC reflex was associated with the performance of animals in the absence and presence of auditory distractors. These results are in line with what we have previously observed, for example, in mice where greater suppression of auditory nerve responses by contralateral noise was associated with better performance in a visual selective attention task with auditory distractors (Terreros et al., 2016).

In the early temporal window, we found that the strength of the MOC reflex (especially at 4080 Hz) predicted the performance of the animals in the presence of auditory distractors (Table 1). In contrast, generalized linear model analyses showed no significant correlation between individual MOC reflex values and animal performance in the late time window (3–5 s) (Table 2). Again, this probably relates with the low number of trials available for analysis. Especially because when we considered only the MOC 4080 Hz reflex value, a linear regression did yield significant effects at day 9 (VOC-1) (Figure 4C). This is supported by the fact that for the ITI-time period (with significantly more trials) the generalized linear model analysis found associations between the MOC reflex and the animals’ responses in the presence of auditory distractors (Table 3). Moreover, these results were similar to those observed in the early period (Figure 4).

Despite the above, it is essential to highlight the limitations of this work. One of them is that the evaluation of the MOC reflex was performed at separate times from the experimental protocol. This implicitly assumes that the MOC reflex can be treated as a single, stable trait, which is not necessarily valid. This limits us from establishing more direct relationships between both tests and leaves open the possibility of having obtained different MOC reflex values in the experimental conditions of the 12-day protocol. However, it is still noteworthy to have performed the measurement of the MOC reflex in fully awake animals, especially considering that evidence shows that strength of the MOC reflex is underestimated in anaesthetized chinchillas (Aedo et al., 2015). Also, and perhaps the most relevant limitation is that our results were obtained from a task that was not designed to assess working memory directly. We had to arbitrarily select late responses from a test initially designed for visual attention to a stimulus (Delano et al., 2007). Without going any further, almost 70% of the lever presses in the response window occurred in less than a second after the target light was turned off and only 5.3% occurred in the window that we defined as late. This explains the fact that our late window had such a low number of responses, and hence all the difficulties that this caused in the data analysis. Moreover, we cannot rule out that lever-related body orientation strategies may be biasing late responses, thus decreasing the working memory load. The same is true for the fact that we used the ITI responses as a proxy for valid late responses. Although it seems reasonable to assume that the animal was responding to the target stimulus (given the results observed in the basal period), the fact that these responses had no reward meant that their interpretation could not be completely equivalent to those that occurred within the valid response period. For the same reason, these experiments should be replicated in tests that are designed exclusively to evaluate WM, for example, by training animals to provide delayed responses with retractable levers to a stimulus. Finally, since we did not perform brain or cochlear function recordings during the task, nor any kind of functional manipulation, our ability to speculate about brain mechanisms is severely limited. For example, even assuming that our results for late responses are due to working memory, we cannot establish the potential neural networks involved (e.g., whether they are linked to visual processing areas, or perhaps to motor or premotor regions). Therefore, we believe that in the future it is necessary to study the brain dynamics associated with this type of behavior or to focus on manipulations that allow intervention of MOC function in behaving animals (e.g., with optogenetics or DREADD tools), in order to be able to dissect underlying mechanisms.

Concerning to visual working memory, our laboratory recently found evidence in humans showing that the MOC reflex is dynamically modulated when relevant visual stimuli are held in mind (Marcenaro et al., 2021). Together, with the present results in chinchillas, we propose a relationship between MOC function and working memory. In addition to our findings, Sörqvist et al. (2012) found modulation of the wave V of auditory brainstem responses during verbal–visual working memory, suggesting that top-down suppression of ascending auditory responses is important for the capacity of filtering distracting stimuli during working memory paradigms (Vogel et al., 2005; Sörqvist et al., 2012; Gaspar et al., 2016). Our results strengthen this notion, including the idea that the efferent function is also a predictor of performance in these tasks.

Therefore, our findings are in agreement with previous evidence that positions the olivocochlear efferent system as part of a dynamic network that is actively regulating sensory inputs as a function of the organism’s relationship with the world and that is sensitive to cognitive states and experience (Oatman, 1971; Delano et al., 2007; Wittekindt et al., 2014; Terreros et al., 2016; Dragicevic et al., 2019; Bowen et al., 2020; Lauer et al., 2021; Marcenaro et al., 2021). This is also supported by anatomical evidence showing that, along with the auditory cortex, other cortical regions may interact directly with the efferent system (Olthof et al., 2019). Therefore, we believe it is pertinent to begin to expand the current understanding of the link between the efferent system and cognitive processes. In this context, the current evidence suggests, at least, this descending network is involved in sensory control associated with working memory, but that it is also possible to extend this notion to cognition as a global phenomenon. Moreover, in general terms, it makes sense to expect that any kind of relevant change in the individual–environment relationship will have consequences on the state of perceptual systems, on the signals entering the organism. Thus, we hypothesize that the auditory efferent control is probably related to the cognitive load of the organism, rather than to specific cognitive functions.

## Data Availability Statement

The raw data supporting the conclusions of this article will be made available by the authors, without undue reservation.

## Ethics Statement

The animal study was reviewed and approved by the Animal Bioethics Committee, Faculty of Medicine, University of Chile.

## Author Contributions

MB, PD, SV-J, and GT: original idea and experimental conceptualization. MB, GB-M, and SV-J: experimental development. GB-M and SV-J: data analysis. SV-J: manuscript writing. GB-M, PD, DM-Z, LR, GT, and SV-J: manuscript editing. PD and GT: project administration and supervision. PD: funding acquisition. All authors contributed to the article and approved the submitted version.

## Conflict of Interest

The authors declare that the research was conducted in the absence of any commercial or financial relationships that could be construed as a potential conflict of interest.

## Publisher’s Note

All claims expressed in this article are solely those of the authors and do not necessarily represent those of their affiliated organizations, or those of the publisher, the editors and the reviewers. Any product that may be evaluated in this article, or claim that may be made by its manufacturer, is not guaranteed or endorsed by the publisher.
